# Clinical Outcomes of Intravascular Lithotripsy in Diabetic Patients with Stent Failure: Insights from the COIL Registry

**DOI:** 10.3390/jcm15218330

**Published:** 2026-10-26

**Authors:** Michał Kuzemczak, Janusz Lipiecki, Jacek Legutko, Johan Bennett, Joseph Dens, Mohammad Alkhalil

**Affiliations:** 1 Division of Emergency Medicine, Poznan University of Medical Sciences, 61-701 Poznan, Poland; michal.kuzemczak@gmail.com; 2 Department of Internal Diseases and Cardiology, Medical University of Warsaw, 02-005 Warsaw, Poland; 3 Department of Interventional Cardiology and Internal Diseases, Military Institute of Medicine—National Research Institute, 05-119 Legionowo, Poland; 4 Centre de Cardiologie Interventionnelle, Pôle Santé République, 63100 Clermont-Ferrand, France; janusz.lipiecki@hotmail.fr; 5 Department of Interventional Cardiology, Institute of Cardiology, Jagiellonian University Medical College, 31-202 Cracow, Poland; jacek.legutko@uj.edu.pl; 6 Clinical Department of Interventional Cardiology, The Saint John Paul II Hospital, 31-202 Cracow, Poland; 7 Department of Cardiovascular Medicine, KU Leuven, University Hospitals Leuven, 3000 Leuven, Belgium; johan.bennett@uzleuven.be; 8 Ziekenhuis Oost-Limburg, 3600 Genk, Belgium; 9 Cardiothoracic Centre, Freeman Hospital, Newcastle-upon-Tyne NE7 7DN, UK; 10 Translational and Clinical Research Institute, Newcastle University, Newcastle-upon-Tyne NE1 7RU, UK

**Keywords:** intravascular lithotripsy, Shockwave, stent failure, in-stent restenosis, diabetes

## Abstract

**Background/Objectives**: Although still considered off-label, there are accumulating data demonstrating that intravascular lithotripsy (IVL) is safe and effective in treating patients with stent failure (SF). Diabetes (DM) is an independent predictor of recurrent SF. Whether IVL has a differential treatment effect depending on the diabetic status of SF patients undergoing IVL has never been studied. Therefore, we aimed to compare IVL outcomes in patients with SF and concomitant DM versus non-DM counterparts. **Methods**: This is a pre-specified subgroup analysis of the COIL registry (coronary intravascular lithotripsy in patients with stent failure), which included patients who underwent IVL for SF, divided according to the patients’ diabetic status. Procedural and clinical outcomes were compared between DM and non-DM patients. The primary clinical endpoint was major adverse clinical event (MACE) defined as the composite of cardiovascular death, spontaneous myocardial infarction, or target vessel revascularization (TVR) at one-year follow-up. **Results**: There were 102 patients enrolled in the coronary intravascular lithotripsy in patients with stent failure (COIL) registry, of whom 47 (46.1%) had DM. Most baseline clinical characteristics were similar between the groups, although patients with DM had higher rates of hypertension and hypercholesterolemia. The use of intracoronary imaging (53% and 56%, respectively, *p* = 0.75) and the procedural success rate (77% vs. 80%, *p* = 0.68) were similar in DM and non-DM patients. No difference was observed in the incidence of in-hospital (2.1% vs. 1.8%, *p* = 0.91) and one-year MACE (12.8% vs. 18.2%, *p* = 0.45). **Conclusions**: In patients undergoing IVL for the treatment of SF, procedural and one-year clinical outcomes did not differ significantly between DM and non-DM patients, although the limited sample size should be acknowledged.

## 1. Introduction

Patients with coronary artery disease (CAD) and concomitant diabetes mellitus (DM) are at significantly higher risk of cardiovascular events compared to their non-diabetic counterparts [1,2]. The heightened cardiovascular risk burdening this subset of patients is multifactorial and stems from an interplay of multiple factors. Insulin resistance, hyperglycemia, deleterious effects of advanced glycation end products (AGEs), and chronic inflammation are key disease components accelerating progression of the disease [1,2,3,4]. This includes not only the formation of de novo coronary lesions, but also progression of the disease in the previously stented segments. The notion is supported by a large body of evidence indicating that DM patients undergoing percutaneous coronary intervention (PCI) are more prone to in-stent restenosis (ISR) [4,5,6]. Furthermore, it has also been shown that the clinical entity is an independent predictor of recurrent ISR [4,6].

Stent failure, most commonly demonstrated by ISR, frequently coexists with coronary artery calcifications (CACs), which have been shown to be associated with worse clinical outcomes secondary to suboptimal lesion preparation, stent delivery failure, and underexpansion [7]. CACs, present in approximately 20% of patients undergoing PCI and more prevalent among DM individuals, have prompted the development of a wide array of calcium-modification devices [8,9,10]. While the repertoire of the modalities dedicated to calcified de novo coronary lesions has expanded considerably in recent years, calcific ISR still constitutes a more demanding target for PCI procedures [11,12].

Among available calcium-modification techniques, intravascular lithotripsy (IVL) has emerged as a treatment modality with high effectiveness and an excellent safety profile [13]. Importantly, accumulating evidence derived from anecdotal reports and multicenter registries indicates that IVL appears to be effective in a wider array of clinical contexts [14,15,16,17,18,19]. Namely, the modality has been found to be effective not only in de novo calcified coronary lesions, but also in cases of SF.

Since DM patients are more prone to CACs along with a higher risk of SF, exploring novel treatment modalities targeting calcific ISR is essential to enhance procedural success and long-term cardiovascular outcomes in this high-risk population. Noteworthy, it remains unknown whether IVL has a differential treatment effect according to DM status in CAD patients affected by SF. To the best of our knowledge, no previous study has specifically compared procedural and clinical outcomes of IVL for SF between patients with and without DM, leaving an important evidence gap in this particularly high-risk population. Therefore, the aim of the present study was to evaluate whether the safety and effectiveness of IVL in patients presenting with SF is influenced by DM status.

## 2. Materials and Methods

This study constitutes a pre-specified subgroup analysis comparing diabetic and non-diabetic patients enrolled in the Coronary Intravascular Lithotripsy in Stent Failure (COIL) registry. The COIL registry is an international, multicenter, retrospective study designed to evaluate clinical outcomes following IVL for SF across six European centers. Between January 2019 and May 2022, the registry included patients presenting with SF unresponsive to conventional balloon angioplasty who were treated with the Shockwave C2 IVL system (Shockwave Medical Inc., Santa Clara, CA, USA). The methodology and baseline characteristics of the overall COIL registry population have been described previously [16]. For the purpose of the present analysis, data on baseline clinical status, procedural characteristics, and follow-up outcomes were collected prospectively using a standardized case report form. All data were anonymized before analysis, and both in-hospital and one-year clinical outcomes were evaluated and compared between DM and non-DM cohorts.

### 2.1. Description of the Procedure

The Shockwave C2 Intravascular Lithotripsy (IVL) system (Shockwave Medical Inc., Santa Clara, CA, USA) is composed of a rapid-exchange balloon catheter integrated with lithotripsy emitters, connected to an external pulse generator [10]. Within the balloon, two emitters spaced 6 mm apart produce circumferential acoustic pressure waves to fracture and modify calcified coronary plaque. Previously available catheters were capable of delivering up to 80 pulses (administered in sets of 10), whereas the next iteration of the device delivers up to 120 pulses. The latter was not used in the present study. Furthermore, the latest device generation designed for improved deliverability (Shockwave C2 Aero IVL catheter, Shockwave Medical Inc., Santa Clara, USA) was also not used since it was not available at that time.

During PCI, pre-dilation using semi-compliant or non-compliant balloons was performed at the operator’s discretion prior to IVL application. The IVL balloon diameter was chosen to match the reference vessel diameter. The IVL treatment involved initial inflation to 4 atm, delivery of up to 10 lithotripsy pulses per cycle, followed by balloon inflation to 6 atm in order to compress the modified plaque. Decisions regarding further lesion preparation and stent implantation strategy were left at the operator’s discretion.

### 2.2. Study Population

The present analysis is based on data from the all-comers COIL registry, which enrolled patients treated with IVL within lesions located in previously stented coronary segments affected by SF. Patients were eligible if they underwent IVL for SF involving a previously stented coronary segment and fulfilled at least one of the following lesion-level criteria: (1) angiographically confirmed ISR with a diameter stenosis of ≥70%, or (2) acute stent underexpansion unresponsive to conventional non-compliant balloon dilation, characterized by a typical “dog-bone” effect during inflation, indicating resistance to expansion. Accordingly, the term SF throughout the present study refers specifically to ISR and resistant acute stent underexpansion rather than other forms of stent-related failure (e.g., stent thrombosis). Eligibility was not restricted by clinical presentation or target vessel. Patients presenting with stable angina, silent myocardial ischemia, acute coronary syndrome, or heart failure symptoms were eligible for inclusion. No additional pre-specified exclusion criteria were applied within the COIL registry beyond the above eligibility requirements. DM status was determined from the documented medical history and, when necessary, supported by available diagnostic laboratory data. DM status was available for all patients included in the COIL registry, and no patients were excluded from the present subgroup analysis due to missing DM data. For the present subgroup analysis, procedural and clinical outcomes—both in-hospital and at one-year follow-up—were compared between DM and non-DM patients. The primary clinical endpoint, pre-specified in the original COIL registry protocol, was major adverse cardiovascular events (MACEs), defined as the composite of cardiovascular death, spontaneous myocardial infarction, or target vessel revascularization at one-year follow-up. Procedural success was defined as residual stenosis < 30% on quantitative coronary angiography in patients who survived the index hospitalization without in-hospital adverse events. All the analyzed data used in the analysis were fully anonymized prior to evaluation.

### 2.3. Coronary Angiogram Analysis

Coronary angiograms obtained before and after IVL were reviewed offline by an independent investigator who was blinded to baseline characteristics (including diabetic status) and clinical outcomes. Quantitative coronary angiography (QCA) was conducted using either the CAAS QCA system (Pie Medical Imaging, Maastricht, The Netherlands) or the QAngio XA 3D/QFR platform (Medis Medical Imaging Systems bv., Leiden, The Netherlands). Measurements were derived from two orthogonal projections selected to avoid vessel overlap and foreshortening, and the mean values were used for the analysis. Baseline QCA parameters comprised reference vessel diameter, minimal lumen diameter (MLD), and percentage diameter stenosis (%DS) before IVL, while final MLD and %DS were determined after completion of the PCI procedure. Severe calcifications were defined as the presence of radiopaque densities without cardiac motion prior to contrast injection and involving both sides of a coronary artery wall [18]. The use of intravascular imaging modalities (IVUS or OCT) was not required for enrollment; however, their application before or after IVL was documented in the case report form. Detailed intracoronary imaging-derived measurements were not available for the analysis.

### 2.4. Statistical Analysis

The normality of continuous variables was examined using the Shapiro–Wilk test. Data conforming to a normal distribution are presented as mean ± standard deviation and were compared using the Student’s *t*-test. Variables with non-normal distributions are reported as median with interquartile range (IQR) and were compared using the Mann–Whitney U test. Categorical variables are expressed as counts and percentages, with differences between groups assessed using the Chi-square test or Fisher’s exact test, as appropriate. Time-to-event outcomes were illustrated using Kaplan–Meier survival curves, and comparisons between DM and non-DM cohorts were performed using the log-rank test. Given the relatively small sample size and limited number of clinical events, no formal adjustment for potential confounding factors was performed. Multivariable adjustment was not performed because the number of events was insufficient to support a reliable multivariable model. Therefore, comparisons between DM and non-DM patients were based on unadjusted analyses. Missing data were handled using an available-case approach, with analyses performed on the available observations for each variable. No imputation of missing values was performed. All statistical analyses were carried out using SPSS Statistics version 28.0 (IBM Corp., Chicago, IL, USA), and a *p*-value < 0.05 was considered statistically significant.

### 2.5. Bioethics

The present study was carried out in accordance with the Declaration of Helsinki. The institutional Medical Ethical Committee waived the requirement for individual informed consent since investigators at each participating center ensured that all patient data were fully anonymized prior to extraction from relevant databases and were normally utilized for routine patient clinical care.

## 3. Results

A total of 102 patients, of whom 47 were diabetic and 55 were non-diabetic, underwent IVL for SF. Patients with DM more often suffered from hypertension (94% vs. 71%, *p* = 0.003). Of the 102 patients, 91 (89.2%) presented with ISR and 11 (10.8%) with acute stent underexpansion. Patients with DM accounted for 18.2% of those with acute stent underexpansion compared with 49.5% of those with ISR (*p* = 0.060), corresponding to a lower frequency of acute stent underexpansion in DM than in non-DM patients (4.3% vs. 16.4%).

Angiographic and procedural characteristics were largely comparable between the two cohorts, although DM patients had fewer ostial lesions (6% vs. 20%, *p* = 0.046), a greater number of stent layers (median 1 [IQR 1–2] vs. 1 [IQR 1–1], *p* = 0.024), a smaller maximum pre-dilation balloon size (3.0 mm [2.8–3.5] vs. 3.5 mm [3.0–3.5], *p* = 0.028), a smaller Shockwave balloon size (3.5 mm [3.0–3.5] vs. 3.5 mm [3.5–4.0], *p* = 0.001), and a smaller maximum post-dilation balloon size (3.5 mm [3.0–3.8] vs. 3.5 mm [3.5–4.1], *p* = 0.029). Procedural success rates were high and similar in DM and non-DM patients (77% vs. 80%, *p* = 0.68), with comparable residual stenosis < 30% (79% vs. 80%, *p* = 0.87). Baseline clinical, angiographic, and procedural characteristics are presented in Table 1 and Table 2.

**Figure 1:**
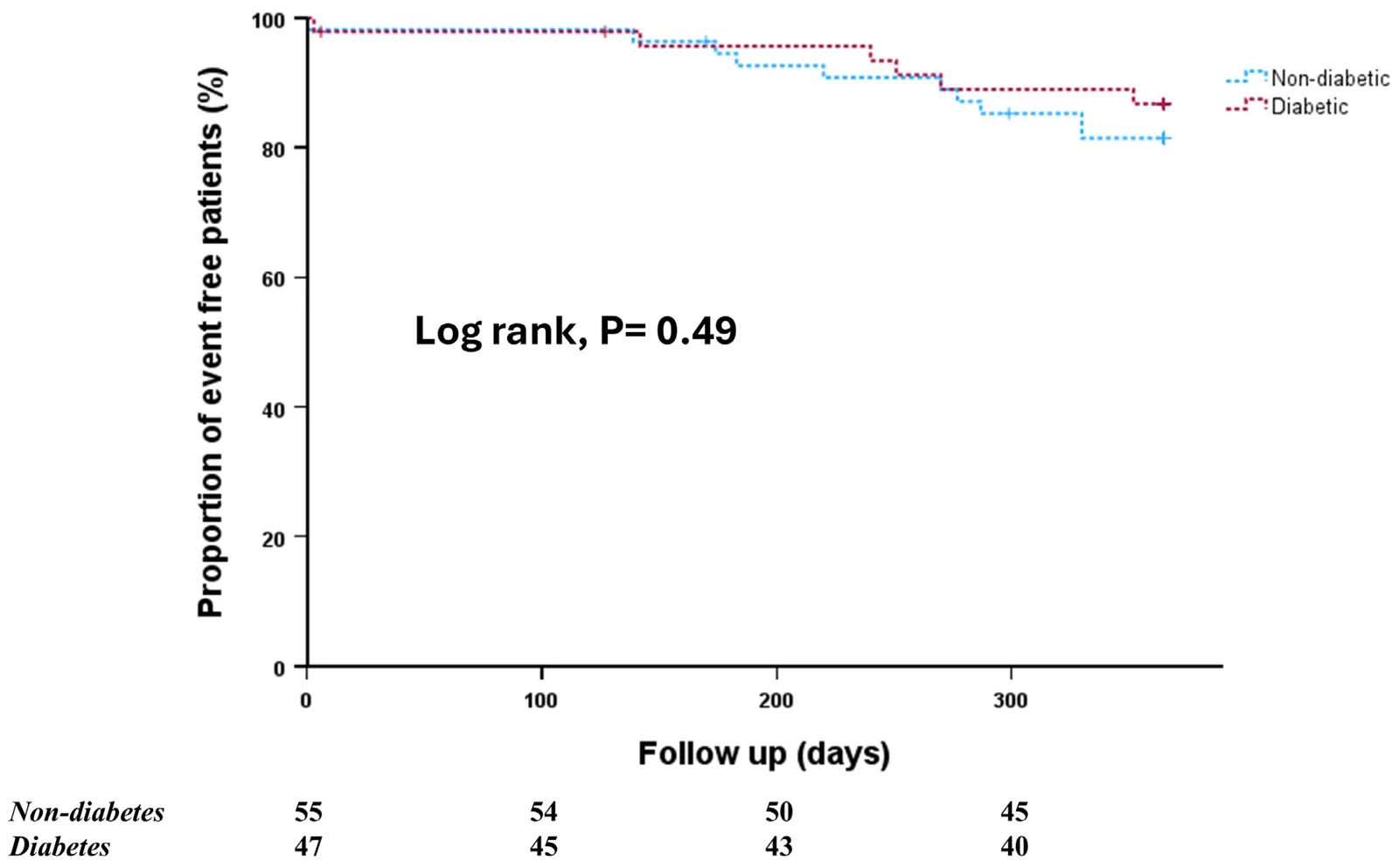
The primary endpoint in patients undergoing intravascular lithotripsy (IVL) within stented segments, according to the patients’ DM status. Kaplan–Meier curves of the cumulative incidence of cardiovascular death, spontaneous myocardial infarction, and target vessel revascularization at one-year follow-up in patients with and without DM.

In-hospital MACE rates were low (2.0% overall), with no difference between the groups. At one-year follow-up, the rate of mortality (6.4% vs. 3.6%, *p* = 0.52), spontaneous myocardial infarction (2.1% vs. 7.3%, *p* = 0.23), target lesion revascularization (6.4% vs. 16.4%, *p* = 0.12), and target vessel revascularization (8.5% vs. 16.4%, *p* = 0.24) did not differ between DM and non-DM patients. Of the five deaths reported during one-year follow-up, one was cardiovascular and occurred in the DM group. The rates of stent thrombosis, non-target vessel revascularization, and composite MACE at one-year follow-up were likewise comparable. Procedural and one-year clinical outcomes following IVL treatment are presented in Table 3. The crude one-year MACE rates were 12.8% in DM and 18.2% in non-DM patients (chi-square *p* = 0.45), whereas a comparison of the corresponding Kaplan–Meier curves using the log-rank test (time-to-event analysis) yielded a *p*-value of 0.49 (Figure 1).

## 4. Discussion

The present study demonstrates a pre-specified subgroup analysis comparing DM and non-DM patients enrolled in the Coronary Intravascular Lithotripsy in Stent Failure (COIL) registry [14]. This is the first study evaluating procedural and clinical outcomes of diabetic real-world patients with SF undergoing IVL within the largest to date multicenter registry. The main findings of the present study can be summarized as follows: /1/IVL is a safe and feasible treatment modality for calcific SF irrespective of patients’ diabetic status; /2/IVL use in patients with SF is associated with similar procedural success rates and acute angiographic results in both DM and non-DM patients; /3/no difference was observed between the groups in one-year clinical outcomes, including MACE, mortality, MI and TVR; and /4/despite still being considered off-label, IVL provides comparable immediate and long-term clinical outcomes across both DM and non-DM cohorts of patients affected by SF.

Previous studies have supported the feasibility of IVL in increasingly complex coronary settings, including stent underexpansion. Evidence from the IVL-DRAGON and CRUNCH registries demonstrated the utility of IVL in this subset of lesions, with procedural/device success rates of 72.6% and 92.3%, respectively, and significant improvement in stent expansion [20,21]. More recently, the BENELUX-IVL registry extended these observations to a broad real-world population that included ISR, aorto-ostial lesions, bifurcations, and chronic total occlusions, with favorable clinical outcomes during 2-year follow-up [22]. Similarly, a meta-analysis of IVL for calcified left main disease reported a pooled procedural success rate of 98.8% with low rates of procedural complications [23]. Together, these data support the use of IVL across a range of complex coronary anatomies and provide a broader context for the present findings in patients with SF.

Despite significant advances in interventional cardiology, SF and its leading cause—ISR—remain the Achilles heel of PCI procedures [24]. Novel devices, better stent designs, and the increasing use of intravascular imaging modalities have reduced, but not eliminated, this PCI-related complication. According to available data, ISR accounts for 5–10% of all PCIs performed in everyday clinical practice, and DM is a strong predictor of ISR onset and recurrence [24,25]. Khalid et al. reported that on follow-up coronary angiography, post-PCI diabetic patients presenting with ISR, compared to non-ISR counterparts, were more symptomatic, had higher HbA1c levels, a longer duration of DM, and had received longer and/or smaller diameter stents during their index PCI [26]. In the present study, neither DM duration nor indices of glycemic control, including HbA1c levels, were systematically collected. Therefore, we were unable to assess whether the duration of DM or the degree of glycemic control influenced response to IVL or clinical outcomes. Data on stent characteristics were also not available. Furthermore, DM and non-DM ISR patients did not differ with regard to clinical manifestation of the disease as reflected by comparable CCS class and percentage of patients with stable angina across studied cohorts. This stays in concordance with the study by Paramasivam et al., which revealed similar clinical manifestations of ISR in DM and non-DM individuals [27].

Both groups were comparable with regard to the majority of baseline characteristics. However, patients without DM were more often affected by ostial lesions (20% vs. 6%). This stays in discordance with available data indicating that DM patients more often suffer from complex lesions, including bifurcations and ostial stenoses [28]. However, the observed discrepancy may stem from a selection bias. Namely, the patients included in the COIL registry may not accurately reflect cohorts of DM and non-DM patients that would be enrolled consecutively from the general population. In the present study, some of the enrolled patients had been scheduled for an elective PCI based on the previously performed coronary angiography. Therefore, it cannot be ruled out that there could have been a significant proportion of DM patients with left main/multivessel diseases and concomitant ostial lesions who were scheduled for CABG instead of PCI. This may have reduced the proportion of ostial lesions in the diabetic cohort of patients in the present study [29]. Importantly, there has been accumulating evidence indicating that ostial lesions are more prone to the incidence of TLR and higher mortality. This applies to both de novo and restenotic lesions [30]. With regard to the latter, data derived from the COIL registry confirmed that ostial restenotic disease targeted by IVL was independently associated with one-year MACE incidence, and the risk was more than 5-fold higher (HR = 5.16) compared to non-ostial restenotic disease [16]. Furthermore, although the distribution of SF phenotypes did not differ significantly between the groups, the potential impact of the different underlying mechanisms of SF on clinical outcomes cannot be excluded.

Interestingly, the present study revealed that restenotic DM patients, despite their unfavorable risk profile (more severe CACs, greater propensity for accelerated atherosclerosis and restenosis), benefited from IVL similarly to their non-DM counterparts. Namely, the present study revealed no significant difference between the studied groups in the incidence of clinical endpoints, including in-hospital and one-year outcomes. This is noteworthy since DM has been identified as an independent predictor of SF, including ISR and its recurrence. Although the present findings do not demonstrate a true ‘equalizing’ effect of IVL (similarly to the previously reported equalization of sex-specific outcomes), the absence of statistically significant differences between DM and non-DM patients may generate the hypothesis that IVL could attenuate some of the adverse impact of DM in patients with SF [31,32]. This interpretation remains speculative and requires confirmation in larger, appropriately powered studies. Feng et al. demonstrated that DM and coronary microvascular disfunction were independent predictors of MACE at 24 months following a PCI procedure with the use of rotational atherectomy (RA) [33]. In this context, the present findings may also raise the hypothesis that IVL could reduce some of the excess cardiovascular risk observed in DM patients undergoing other calcium-modification strategies, such as RA. However, direct comparisons cannot be made from the present study. In ISR patients, the magnitude of IVL-dependent risk equalization may be even more pronounced since RA of underexpanded stents (one of the most frequent mechanisms of ISR) is more risky than RA of calcified de novo lesions [34]. Therefore, as stated in the European expert consensus, it is highly desirable to perform RA within calcified lesions concomitant with underexpanded stents with extreme caution and by highly skilled operators [34]. Accumulating data to date indicate that IVL may serve as a straightforward alternative to RA and can be a first-line approach in such clinical instances (provided that a particular lesion is crossable).

In the study by Feng et al., Kaplan–Meier curves demonstrating clinical events in DM and non-DM patients started to diverge one year following the RA procedure and, even more importantly, the difference between the groups became more significant as the follow-up increased [33]. Paramasivam et al. reported no significant difference in one-year outcomes (composite of death, myocardial infarction, and repeat-target lesion revascularization) between DM and non-DM patients presenting with ISR [27]. Therefore, it cannot be ruled out that in the present study, longer than one-year follow-up would reveal significant differences between the two studied cohorts. However, this potential assumption seems to be challenged by Zhao et al., indicating similar two-year outcomes in DM and non-DM patients undergoing repeat second-generation DES implantation for DES-ISR [35]. In order to elucidate all the above-mentioned points, more studies evaluating IVL for ISR with longer follow-up periods are warranted.

Currently, apart from IVL, there is a plethora of other catheter-based techniques for the treatment of ISR, including another DES implantation, vascular brachytherapy, excimer laser atherectomy (ELCA), and drug-eluting balloons (DEBs), but comparisons of the techniques in ISR patients with co-existing DM are scarce [24,36]. In the DM-Dragon registry evaluating DEB vs. DES for ISR in DM patients, no significant difference in TLR between unselected cohorts of DM and non-DM patients was observed at 3-year follow-up, which also remained non-significant following propensity-score matching (PSM) [37]. The rates of MI and all-cause death were significantly higher among unselected DM patients, but the differences became insignificant after PSM. Further studies comparing different approaches to ISR in diabetic vs. non-diabetic patients are needed since the findings accumulated to date should be considered hypothesis-generating.

Several limitations of the present study need to be acknowledged when interpreting the results. Firstly, the COIL registry was retrospective in nature and therefore subject to limitations inherent to observational studies, including potential selection bias, residual confounding, and heterogeneity in procedural decision-making across participating centers. Although the subgroup analysis was pre-specified, the retrospective design precludes complete control for unmeasured confounders that may have influenced procedural or clinical outcomes. Secondly, the relatively small sample size limited the statistical power to detect modest differences between the groups and increased the possibility of type II error. The limited number of clinical events also restricted the feasibility of more extensive multivariable adjustment and additional subgroup analyses. Given the above, the absence of statistically significant differences between DM and non-DM patients should be interpreted cautiously and should not be considered definitive evidence of equivalence between the groups. Thirdly, data on stent platforms implanted during the index procedure were not available; therefore, possible associations between different stent types and IVL outcomes cannot be studied. Furthermore, medical adherence to guideline-directed regimens in the studied groups was not evaluated. This makes it difficult to ascertain whether potential variations in medical management, including adequate glycemic control, affected the outcomes. In addition, data on DM duration and HbA1c levels were not systematically available, precluding evaluation of whether the disease duration or metabolic control modified the response to IVL or influenced long-term outcomes. Furthermore, detailed characterization of DM and glucose-lowering therapy was not available. This may be relevant given a large body of evidence linking poor glycemic control with ISR [26,38,39]. Furthermore, ISR is an umbrella term with multiple underlying mechanisms (mechanical and biological, with various intra-stent, extra-stent, and stent-related factors involved), sometimes occurring simultaneously, which may influence clinical response to different PCI interventions and long-term outcomes [24]. In the present study, intravascular imaging (IVUS or OCT) was not systematically performed or recorded [17]. Detailed intravascular imaging results, crucial when performing calcium debulking/modifying techniques, would provide important insights into underlying mechanisms and patterns of calcific ISR, and could help to describe factors determining IVL outcomes in this subset of patients [40]. This would shed more light on the role of a differential approach to ISR depending on intravascular imaging appearances (e.g., ISR due to stent underexpansion secondary to coronary calcifications vs. calcified neoatherosclerotic ISR) and contribute toward a more personalized medicine paradigm based on ISR tissue patterns [24]. A comprehensive assessment of non-cardiovascular comorbidity burden was not systematically available in the registry. Therefore, the potential influence of unmeasured comorbid conditions on long-term outcomes cannot be excluded. Finally, the follow-up period was limited to one year, which is particularly relevant given that differences in recurrent ISR or other late adverse events may become apparent beyond the first year after PCI.

## 5. Conclusions

The present study indicates that IVL use for the treatment of SF was associated with similar procedural and one-year clinical outcomes in DM and non-DM patients. However, given the limited sample size and number of clinical events, these findings should be interpreted with caution. Further studies with larger populations, longer follow-up periods, and systematic intravascular imaging are warranted to corroborate our findings, and to explore potential relationships between DM and long-term effects of IVL in the subset of SF patients. This would help to establish a more individualized approach to SF therapy, depending on lesion characteristics and patients’ comorbidities, including DM.

## Figures and Tables

**Table 1:** Baseline clinical characteristics of the included patients.

	Whole Cohort (n = 102)	Non-Diabetic (n = 55)	Diabetic (n = 47)	*p*-Value
Age _(mean±SD)_	73 ± 9	73 ± 9	72 ± 8	0.48
Male gender _(n, %)_	83 (81%)	46 (84%)	37 (79%)	0.53
Body mass index _(mean±SD)_	28 ± 5	28 ± 4	29 ± 5	0.28
Stable angina _(n, %)_	52 (51%)	26 (47%)	26 (55%)	0.42
Canadian Cardiovascular Society angina Grade _(median, IQR)_	3 (2–3)	3 (1–3)	3 (2–3)	0.19
Hypertension _(n, %)_	83 (81%)	39 (71%)	44 (94%)	0.003
Current smoking _(n, %)_	8 (8%)	3 (6%)	5 (11%)	0.33
Hypercholesterolemia _(n, %)_	71 (70%)	32 (58%)	39 (83%)	0.007
Previous MI _(n, %)_	81 (79%)	41 (75%)	40 (85%)	0.19
Previous CABG _(n, %)_	14 (14%)	8 (15%)	6 (13%)	0.80
Previous stroke _(n, %)_	9 (9%)	5 (9%)	4 (9%)	0.92
Previous pacemaker _(n, %)_	7 (7%)	4 (7%)	3 (6%)	0.86
Creatinine clearance _(median, IQR)_	64 (46–87)	63 (46–84)	65 (48–90)	0.70
LV ejection fraction _(median, IQR)_	50 (39–55)	50 (43–55)	48 (37–55)	0.35
Timing from previous stent implantation (months) _(median, IQR)_	24 (7–76)	24 (7–75)	14 (7–80)	0.76
In-stent restenosis (n, %)	91 (89.2%)	46 (83.6%)	45 (95.7%)	0.06
Acute stent underexpansion (n, %)	11 (10.8%)	9 (16.4%)	2 (4.3%)	0.06

CABG: coronary artery bypass graft, LV: left ventricle, MI: myocardial infarction.

**Table 2:** Angiographic and procedural characteristics.

	Whole Cohort (n = 102)	Non-Diabetic (n = 55)	Diabetic (n = 47)	*p*-Value
Radial access _(n, %)_	85 (83%)	44 (80%)	41 (87%)	0.48
Left main/LAD _(n, %)_	45 (44%)	23 (42%)	22 (47%)	0.61
Ostial lesion _(n, %)_	14 (14%)	11 (20%)	3 (6%)	0.046
Bifurcation _(n, %)_	24 (24%)	13 (24%)	11 (23%)	0.98
Severe calcification _(n, %)_	62 (61%)	29 (53%)	33 (70%)	0.23
Number of stent layers _(median, IQR)_	1 (1–2)	1 (1–1)	1 (1–2)	0.024
Reference vessel diameter (mm) _(mean±SD)_	3.22 ± 0.57	3.27 ± 0.59	3.16 ± 0.53	0.33
Pre-dilation _(n, %)_	94 (93%)	50 (93%)	44 (94%)	0.84
Maximum pre-dilation balloon size (mm) _(median, IQR)_	3.0 (3.0–3.5)	3.5 (3.0–3.5)	3.0 (2.8–3.5)	0.028
Minimal lumen diameter (pre IVL) (mm) _(mean±SD)_	1.14 ± 0.60	1.17 ± 0.58	1.11 ± 0.63	0.66
Degree of stenosis (pre IVL) (%) _(mean±SD)_	67 ± 20	67 ± 18	67 ± 22	0.88
Lesion length (mm) _(mean±SD)_	16.8 ± 11.2	17.3 ± 12.4	16.3 ± 9.7	0.66
Shockwave balloon size (mm) _(median, IQR)_	3.5 (3.0–4.0)	3.5 (3.5–4.0)	3.5 (3.0–3.5)	0.001
Number of shockwave pulses _(median, IQR)_	80 (65–80)	80 (70–80)	80 (60–80)	0.85
Post-dilation _(n, %)_	50 (49%)	30 (55%)	20 (43%)	0.23
Maximum post-dilation balloon size (mm) _(median, IQR)_	3.5 (3.0–4.0)	3.5 (3.5–4.1)	3.5 (3.0–3.8)	0.029
Total stent length (mm) _(mean±SD)_	29 ± 15	30 ± 14	28 ± 16	0.56
Minimal lumen diameter (final) (mm) _(mean±SD)_	2.53 ± 0.59	2.61 ± 0.60	2.43 ± 0.58	0.15
Degree of stenosis (final) (%) _(mean±SD)_	20 ± 11	20 ± 11	20 ± 12	0.93
Imaging use _(n, %)_	56 (55%)	31 (56%)	25 (53%)	0.75
Fluoroscopy time (minutes) _(mean±SD)_	20.2 ± 9.6	21.5 ± 10.6	18.7 ± 8.3	0.19
Air Kerma (mGy) _(mean±SD)_	1140 ± 836	1220 ± 856	1037 ± 811	0.34
Contrast volume (ml) _(mean±SD)_	166 ± 65	173 ± 67	159 ± 64	0.29
Total procedural time (minutes) _(mean±SD)_	84 ± 35	88 ± 27	80 ± 44	0.34

IVL: intravascular lithotripsy, LAD: left anterior descending artery.

**Table 3:** Procedural and one-year clinical outcomes following IVL treatment.

	Whole Cohort (n = 102)	Non-Diabetic (n = 55)	Diabetic (n = 47)	*p*-Value
Residual stenosis < 30% _(n, %)_	81 (79.4%)	44 (80%)	37 (79%)	0.87
Procedural success _(n, %)_	80 (78.4%)	44 (80%)	36 (77%)	0.68
In-hospital major adverse cardiovascular events _(n, %)_	2 (2.0%)	1 (1.8%)	1 (2.1%)	0.91
One-year outcome				
Death _(n, %)_	5 (4.9%)	2 (3.6%)	3 (6.4%)	0.52 HR 1.82 (0.30–10.89)
Spontaneous myocardial infarction _(n, %)_	5 (4.9%)	4 (7.3%)	1 (2.1%)	0.23 HR 0.29 (0.03–2.59)
Target vessel revascularization _(n, %)_	13 (12.7%)	9 (16.4%)	4 (8.5%)	0.24 HR 0.50 (0.15–1.62)
Target lesion revascularization _(n, %)_	12 (11.8%)	9 (16.4%)	3 (6.4%)	0.12 HR 0.38 (0.10–1.39)
Stent thrombosis _(n, %)_	2 (2%)	2 (3.6%)	0	0.19 HR 0.02 (0–1600.60)
Non-targeted vessel revascularization _(n, %)_	15 (14.7%)	9 (16.4%)	6 (12.8%)	0.61 HR 0.88 (0.31–2.53)
Major adverse cardiovascular events _(n, %)_	16 (15.7%)	10 (18.2%)	6 (12.8%)	0.45 HR 0.78 (0.32–1.91)

## Data Availability

The raw data supporting the conclusions of this article will be made available by the authors on request.

## Abbreviations

The following abbreviations are used in this manuscript:

ACS	Acute coronary syndrome
AGEs	Advanced glycation end products
BMS	Bare metal stents
CABG	Coronary artery bypass graft surgery
CAC	Coronary artery calcification
COIL	Coronary Intravascular Lithotripsy in Stent Failure Registry
CRT	Cardiac resynchronization therapy
DEB	Drug-eluting balloon
DES	Drug-eluting stent
DM	Diabetes mellitus
ELCA	Excimer laser coronary atherectomy
ICD	Implantable cardioverter defibrillator
ISR	In-stent restenosis
IVL	Intravascular lithotripsy
LAD	Left anterior descending artery
MACE	Major adverse cardiovascular event
MI	Myocardial infarction
OCT	Optical coherence tomography
PCI	Percutaneous coronary intervention
RA	Rotational atherectomy
SF	Stent failure
